# Development and Validation of an Instrument to Assess the Cognitive, Behavioral, and Environmental Factors Related to Sodium Intake in Adult Canadians: The Behavioral Assessment Instrument for Dietary Sodium

**DOI:** 10.1016/j.cdnut.2025.107592

**Published:** 2025-10-30

**Authors:** Rola Al Ghali, Michael Prashad, Wendy Lou, JoAnne Arcand

**Affiliations:** 1 Health Sciences, Ontario Tech University, Oshawa, ON, Canada; 2 Dalla Lana School of Public Health, University of Toronto, Toronto, ON, Canada

**Keywords:** survey development, survey validation, sodium, Canadian adults, knowledge, attitudes, behaviors, cognitive, behavioral, environmental factors, BAIS

## Abstract

**Background:**

High sodium intake is a major risk factor for noncommunicable diseases, with most Canadians exceeding recommended levels. Population-wide sodium-reduction interventions include monitoring sodium knowledge, attitudes, and behaviors as a core activity. However, there is a lack of developed instruments incorporating qualitative research and behavioral theories, accompanied by robust validation methods and psychometric testing.

**Objectives:**

This study aimed to develop and validate a comprehensive instrument, the Behavioral Assessment Instrument for dietary Sodium (BAIS), assessing cognitive, behavioral, and environmental influences on sodium intake among Canadian adults.

**Methods:**

Item development was informed by a prior national sodium survey, a Canadian qualitative study, relevant literature, and behavioral theories. Content validity was assessed by 11 experts over 2 rounds and evaluated by computing the content validity index (CVI) for individual items and scale average (S-CVI/Ave). Face validity was verified through one-on-one interviews with 10 Canadian adults (>18 y). Item response theory (IRT) assessed knowledge items, whereas exploratory factor analyses (EFA) and confirmatory factor analyses (CFA) validated the remaining constructs (*n =* 3236; 51.2% women; mean age 49.5 ± 17.7 y). Reliability analyses included intraclass correlation coefficients (ICC) for ordinal questions, Gwet’s first-order agreement coefficient (AC1) for binary questions, and Cronbach’s alpha.

**Results:**

Expert review led to iterative revisions, with round 2 achieving S-CVI/Ave = 1, indicating high content validity. Cognitive interviews (50% male, 23–62 y) confirmed item clarity. IRT results showed acceptable difficulty and discrimination of knowledge variables. EFA and CFA supported a 6-factor structure: Nutrition labeling behaviors, Hedonic influences, perceived control and self-efficacy, priority and concern, delayed concern, and sodium-reduction practices. Internal consistency ranged from acceptable to excellent. ICC was moderate for most ordinal variables, and AC1 was moderate to very good.

**Conclusions:**

The BAIS represents the first validated Canadian instrument to assess multidimensional, theory-based drivers of sodium intake, supporting improved surveillance, policy development, and targeted interventions.

## Introduction

In Canada and globally, high sodium intake is a well-established risk factor for hypertension, cardiovascular disease, stroke, renal disease, and mortality [[1], [2], [3], [4]]. However, most Canadians and people worldwide exceed recommended levels [5,6]. In Canada, lowering sodium intake below 2300 mg/d is estimated to reduce hypertension prevalence by 30% and save the healthcare system CAD $1.38 billion annually [7]. Population-wide sodium-reduction strategies have been implemented to address the health and economic burden of excess sodium, combining upstream policy measures with downstream strategies to influence individual behaviors. Canada’s Sodium-Reduction Strategy and the WHO’s “best buys” for noncommunicable disease prevention call for a combination of voluntary sodium targets for industry, food procurement guidelines, front-of-package labeling, and public education campaigns [8,9].

The WHO emphasizes monitoring and surveillance as critical aspects of population-wide sodium-reduction strategies, including health outcomes, food reformulation, sodium knowledge, attitudes, and behavior (KAB) [9,10]. Several instruments are available to assess sodium KAB globally, including those developed by the Pan American Health Organization/WHO, the Centers for Disease Control and Prevention, and various national health surveillance systems. Some studies have used instruments that are investigator-generated, tailored for specific populations and settings [[11], [12]]; an approach also applied in Canada [[13], [14], [15], [16]]. Although these tools have generated valuable insights, they often lack rigorous theoretical grounding and validation.

Few existing instruments to monitor sodium KAB have employed qualitative research or been guided by behavioral theory [14], limiting their ability to capture the nuanced perspectives and experiences of individuals, as well as the broader set of theoretically derived factors influencing sodium intake behaviors such as perceived risk, perceived control, hedonics, social norms, and motivation [17]. Instead, most instruments narrowly focus on specific cognitive and behavioral factors (e.g., label reading, discretionary salt use), without considering the broader environmental (e.g., food access) and policy contexts. Furthermore, validation of these tools is typically limited to content and face validity assessments, with face validity rarely including robust methods such as cognitive interviewing approaches [18]. As a result, current tools may fail to fully reflect the complexity of sodium-related behaviors or detect change in response to public health interventions. Thus, there is growing recognition that sodium KAB assessment must evolve to better incorporate theory alongside rigorous validation and psychometric testing. Instruments designed accordingly will enhance our understanding of sodium-related behaviors and support population-wide sodium-reduction efforts. Therefore, the first objective of this study was to use qualitative research and behavioral theory to develop and validate (content and face validity) the Behavioral Assessment Instrument for dietary Sodium (BAIS), assessing cognitive, behavioral, and environmental determinants of sodium intake among Canadian adults. The second objective was to explore the underlying constructs of the BAIS through psychometric analyses to confirm dimensionality, construct validity, and reliability.

## Methods

### Study design

This multiphase instrument validation study employed both quantitative and qualitative methods to develop the BAIS. Research activities were conducted across 3 phases. The first objective, development and validation of the BAIS, was addressed in phase A (instrument development) and phase B (content and face validation). The second research objective, psychometric evaluation, was achieved in phase C (construct validity, dimensionality, and reliability assessment). Written informed consent was obtained from all participants prior to data collection, and no identifying data were collected. The study was approved by the Ontario Tech University Research Ethics Board (REB# 17207 and # 17546).

### Phase A: instrument development

#### Frameworks used in instrument development

Instrument development was guided by key health behavior theories, including social cognitive theory (SCT), health belief model (HBM), theory of planned behavior (TPB), and social marketing (SM) principles. Given the complex nature of sodium intake, drawing on multiple frameworks supported a nuanced understanding of its cognitive, behavioral, and environmental determinants. Each theory-informed item selection and framing. SCT guided questions on self-efficacy, observational learning, and environmental facilitators, reflecting the interaction of personal (cognitive), environmental, and behavioral factors [17]. HBM shaped questions on perceived susceptibility, severity, benefits, barriers, cues to action, and confidence to change [17]. TPB informed questions on attitudes, subjective norms, and perceived behavioral control; key drivers of behavioral intention [17]. Although not a behavior change theory, SM’s “4Ps” (Product, Price, Place, and Promotion) guided questions on perceived value, cost, access, and communication of sodium-reduction strategies, emphasizing contextual factors influencing real-world behaviors and decision-making [19].

#### Instrument question generation

Development of the BAIS questions combined inductive and deductive approaches [18]. First, a 2013 national Canadian sodium KAB survey by Arcand et al. [13] served as the foundation, with expert review used to select relevant questions and propose new items or topics. Second, findings from a qualitative study on Canadian adults’ experiences and perceptions of sodium were mapped to constructs from SCT, HBM, TPB, and SM to guide item development [20]. Finally, a targeted literature review identified additional questions from Canadian and international sodium KAB studies and theory-based research on eating and health-related behaviors, supplemented by a recent scoping review of sodium KAB instruments in the Americas [12].

### Phase B: content and face validation

#### Assessment of content validity by experts

A panel of 11 experts in nutrition, sodium, public health, and survey design assessed the BAIS’s content validity over 2 rounds, 7 in the first and 4 in the second [21]. Content validity was evaluated both before and after face validity assessment. Experts rated each item for clarity and construct representativeness, using a 4-point Likert scale and provided written feedback [22,23]. The content validity index (CVI) was calculated at both the item (I-CVI) and survey (S-CVI) levels, with thresholds of ≥0.78 and ≥0.90, respectively [21,23]. I-CVI was calculated as the number of experts rating an item 3 or 4 divided by the total number of experts; average scale-level CVI (S-CVI/Ave) was the mean of all I-CVIs [21,22]. To assess inter-rater agreement, modified kappa statistic was used, and was classified as excellent or good based on the number of experts: for 7 experts, ≥0.85 and ≥0.65 indicated excellent and good agreement, respectively; for 4 experts, the corresponding thresholds were 1.00 and ≥0.67 [21]. Experts also evaluated the comprehensiveness of each construct to ensure all relevant aspects were addressed.

#### Assessment of face validity by Canadian adults

Face validity was assessed through 2 rounds of cognitive interviews with 10 Canadian adults (*n =* 5/round) selected to ensure a sample with diverse sex, age, ethnicity, education level, province, and sodium-reduction behaviors [18,24]. Participants were recruited via social media and met the following criteria: aged 18 or older; able to read and write in English; with internet access and a digital device; and residing in Canada. All cognitive interviews were conducted in English using a video conferencing platform (Zoom). Cognitive interviews used think-aloud and verbal probing techniques to assess participants’ understanding, interpretation, and response processes [24,25]. A semistructured interview guide facilitated the sessions, which were documented using handwritten notes for each question. Findings were compiled and reviewed, then used to revise questions after each round. Interviews continued until no further modifications were needed [24,25]. Participants received an incentive valued at CAD $30 in the form of a gift card as a token of appreciation for their time and feedback.

The instrument was originally developed in English and subsequently translated into French following Leger Marketing’s standardized translation protocol. A native French translator completed the initial translation, which was reviewed and revised by a second translator. Cultural and linguistic adaptations were made to ensure equivalence, particularly for idiomatic expressions and nutrition/public health terminology reflective of the language used by French-speaking Canadians. After translation, face validity testing was conducted among 5 bilingual participants residing in both English- and French-speaking communities to confirm clarity, comprehension, and cultural appropriateness of the instrument items in both languages. Final revisions and quality control were completed by Leger’s team.

#### Instrument piloting

After content and face validity, the instrument was piloted in 2 stages. An initial in-house pilot with 5 members of the research team who assessed item clarity and completion time. Feedback was used to refine the instrument. A second pilot was done by Leger Marketing among 47 participants (26 English-speaking and 21 French-speaking) recruited to reflect diverse sociodemographic characteristics, including language, province, age group, and sex. Participants were invited to provide feedback on the instrument. The feedback was consistently positive, indicating that the questions were perceived as clear, relevant, and well-constructed in both English and French, thereby supporting the face validity of the BAIS.

### Phase C: construct validity, dimensionality, and reliability assessment

#### Instrument administration and assessment of dimensionality and construct validity

The BAIS also underwent dimensionality and construct validity testing. Given the combination of binary (knowledge) and ordinal items, item response theory (IRT) was applied to binary items, whereas exploratory factor analyses (EFA) and confirmatory factor analyses (CFA) were conducted on ordinal items [[26], [27], [28]]. A sample of 1000–1100 participants/analysis was deemed sufficient based on a 10:1 participant-to-variable ratio for factor analysis (87 ordinal variables) and accounting for 20%–25% missing data [13,29]. Between October and November 2024, Leger Marketing recruited 3409 Canadian adults aged 18+, able to read and write in English or French, with internet and device access. Participants were recruited through Leger’s proprietary internet panel of over 400,000 Canadian adults, extensively profiled on sociodemographic characteristics. Stratified random sampling, based on 2021 Statistics Canada census distributions, ensured representativeness across regions, genders, and age groups. Sample quotas were adjusted during fielding to achieve targets. We achieved an 82.0% overall response rate. To maximize data quality, the instrument platform required all questions to be answered, except those with skip logic. Participants were incentivized through Leger’s LEO program (1000 points = CAD $1), receiving CAD $2.50 for the first instrument and CAD $5.00 for the second (test–retest), redeemable for rewards.

After applying quality checks, including the exclusion of participants who answered 2 of Leger’s quality control questions incorrectly and those who completed the survey in ≤12 min, 3236 participants remained: 51.2% women; mean age 49.9 ± 17.7 y; 74.8% White; 37.1% from Ontario; 49.0% urban residents; 37.3% held a bachelor’s degree or higher; and 66% reported annual incomes ≤CAD $99,000. Because factor analysis is sensitive to missing data, only variables with <5% missingness were retained, yielding 2490 complete cases and 48 variables for factor analysis. Knowledge questions had no missing data and were analyzed in the full sample (*n* = 3236; 11 variables).

The sample was randomly split for EFA and CFA (*n =* 1245 each) [18,30]. Sampling adequacy was assessed using the Kaiser–Meyer–Olkin test (≥0.7) and Bartlett’s test of sphericity (*P* < 0.05) [27,31]. To identify the factor structure that best explains the relationships between variables, maximum likelihood estimation was used, guided by parallel analysis, eigenvalues >1, and scree plot inspection, with oblique rotation assuming factor correlation [27,32,33]. Items with loadings <0.4, cross-loadings <0.2 apart, or secondary loadings ≥0.3 were removed. Factors were named based on theoretical alignment [27]. To confirm dimensionality, CFA was performed, using root mean square error of approximation (RMSEA ≤ 0.06), Tucker–Lewis index (TLI ≥ 0.90), comparative fit index (CFI ≥ 0.90), and standardized root mean square residual (SRMR ≤ 0.08) [33,34]. To improve model fit, items with loadings <0.5 were removed, and high-impact correlated error terms were added. Knowledge items (scored 1 = correct, 0 = incorrect) were analyzed using a 2-parameter logistic IRT model selected based on the Akaike Information Criterion. This model estimates item discrimination (a), indicating how well an item differentiates knowledge levels, and difficulty (b), reflecting item challenge with difficulty typically ranging from −3 (easiest) to +3 (most difficult), and discrimination categorized from none (0) to perfect (approaching infinity) [26,28]. IRT analyses used the full sample, split equally for exploratory and confirmatory analysis. Model fit was evaluated using CFA-aligned metrics [33].

#### Reliability assessment

A random subsample of 100 participants completed the instrument for a second time within 2 wk to assess test–retest reliability [35]. Reliability was evaluated using both test–retest and internal consistency metrics. Intraclass correlation coefficients (ICC) were calculated using a single-measure, absolute agreement, 2-way mixed-effects model, with values interpreted as: <0.5 (poor), 0.5–0.75 (moderate), 0.75–0.9 (good), and >0.9 (excellent) [36]. For dichotomous knowledge questions, Gwet’s first-order agreement coefficient, with thresholds of ≤0.20 (poor), 0.21–0.40 (fair), 0.41–0.60 (moderate), 0.61–0.80 (good), and 0.81–1.0 (very good) [37]. Internal consistency was evaluated using Cronbach’s alpha, with values >0.7 indicating acceptable reliability [33,38].

### Instrument coding and statistical analyses

Knowledge was measured using multiple-choice questions coded as correct or incorrect. One question asked participants to identify medical conditions linked to high sodium intake; those correctly identifying ≥8 of 12 conditions were classified as having good knowledge (1), otherwise poor knowledge (0). All other constructs (e.g., attitudes, behaviors, and self-efficacy) were assessed using 5-point Likert scales (1 = not at all important/strongly disagree; 5 = extremely important/strongly agree), with higher scores indicating stronger support for sodium reduction. Negatively worded items were reverse-coded. Descriptive statistics (mean, SD, frequencies, percentages) summarized responses. Analyses were conducted in RStudio (version 2022.12.0).

## Results

The final version of the BAIS included 32 questions (98 items) spanning 12 constructs. After psychometric testing, 13 questions with 34 items were retained across 7 factors: Knowledge, Nutrition Labeling Behaviors, Hedonic Influences, Perceived Control and Self-Efficacy, Priority and Concern, Delayed Concern, and Sodium-Reduction Practices, representing the abbreviated version of the BAIS (BAIS-ab). Detailed results are presented below.

### Phase A: instrument development

Experts identified 24 items from the 2012 national Canadian sodium KAB survey for inclusion [13]. Additional questions were adopted or adapted from existing tools [[39], [40], [41], [42], [43], [44], [45], [46], [47], [48], [49], [50], [51], [52]] or newly developed based on findings from the qualitative study and selected theoretical frameworks [20]. The first version of the BAIS comprised 35 questions, many with multiple subitems, spanning 12 constructs.

### Phase B: content and face validation

Experts (*n* = 7) rated item clarity and representativeness, yielding high item-level CVIs (I-CVI = 0.71–1.00) and S-CVI/Ave of 0.95. Inter-rater agreement ranged from 0.65 to 1.00, indicating good to excellent reliability. Items with low CVIs were removed. On the basis of feedback, questions were removed, revised, or added. The revised instrument included 32 questions, many multi-itemed, across 10 constructs: knowledge, behaviors, attitudes, concern/perceived risk, hedonic factors, cost, social influences, environmental/contextual factors, perceived control, and self-efficacy. This version underwent face validity assessment. Interviewees generally understood and interpreted the questions as intended, with minor revisions made to improve clarity. Suggested edits included adding a “not applicable” option to questions on social and environmental factors; inserting “general” in the question “How do you rate your knowledge related to dietary sodium”; and replacing “and” with “and/or” in “family members, relatives, and friends.” Questions and items were refined and retested until no further changes were required. The French version also demonstrated excellent face validity. In a final round of content validity assessment by 4 experts, all questions and items received an I-CVI of 1.0, resulting in an S-CVI of 1.0, and inter-rater agreement was rated excellent. The total number of questions and items in the BAIS remained unchanged (Supplemental Material).

### Phase C: construct validity, dimensionality, and reliability assessment

#### EFA and CFA on ordinal variables

Of the 32 multi-item questions, 26 were included in dimensionality analyses. Six were excluded based on content; for example, the behavior construct included 2 questions on reasons for limiting or not limiting sodium, plus a binary question on reduction motivators, which were unsuitable for EFA. Sampling adequacy was excellent (Kaiser–Meyer–Olkin = 0.93; range: 0.71–0.96), and Bartlett’s test was significant, confirming data suitability for factor analysis. Initial EFA, based on parallel analysis, scree plot inspection, and eigenvalues >1, suggested a 9-factor solution. However, 3 factors contained only 1 or 2 items, insufficient to represent latent constructs [32]. As a result, models with 8, 7, and 6 factors were tested. The 8- and 7-factor solutions also yielded bivariate factors. As a result, the 6-factor solution was selected. The initial model fit was acceptable: CFI = 0.92, TLI = 0.86, RMSEA = 0.048 [confidence interval (CI): 0.047, 0.050], Bayesian Information Criterion = −2741.87, RMSR = 0.03. Seventeen items with low factor loadings (<0.4) or problematic cross-loadings were excluded, leaving 31 variables. For instance, 2 items: “Engagement in sodium reduction” and “Can cook from scratch,” were excluded due to cross-loadings with <0.2 difference. Another item, “Do not like salty food,” was removed due to a high secondary loading (≥0.3). The final 6-factor model showed improved fit: CFI increased to 0.94, TLI to 0.90, Bayesian Information Criterion lowered to −817.89; RMSEA = 0.052 (CI: 0.049, 0.055), and SRMR = 0.03. The model explained an acceptable 49% of cumulative variance across retained factors (Table 1).

**TABLE 1:** Factor loading results of the final EFA 6-factor model for the abbreviated BAIS (BAIS-ab).

EFA identified latent factors with related variables		Factor loading
Factor 1: nutrition labeling behaviors		
1	Look at the total amount of sodium (mg) on the Nutrition Facts Table	0.88
2	Look at the % Daily Value (% DV) on the Nutrition Facts table	0.81
3	Look for a symbol or a logo on the food package that suggests a product is a healthy choice	0.55
4	Look for a message or claim on the food package that says a product is “low sodium,” “reduced sodium” or “sodium free”	0.60
5	Look at the ingredients list for sodium sources	0.69
6	Read the Nutrition Facts table to determine if a product is high or low sodium	0.56
7	I am willing to invest time to read food labels to find lower sodium foods	0.46

Factor 2: hedonic influences		
1	Sodium makes food taste good	0.56
2	I often have cravings for salty foods	0.79
3	When I have feelings of stress or sadness, I comfort myself by eating foods that may be high in sodium	0.56
4	I enjoy eating salty foods or snacks	0.80

Factor 3: perceived control and self-efficacy		
1	I can control the amount of sodium I consume	0.74
2	Limiting my sodium intake is up to me	0.51
3	If I wanted to, it would be easy for me to limit the sodium in my diet	0.77
4	I have willpower to reduce my sodium intake	0.56
5	I am confident I can identify the sources of sodium in my diet	0.43
6	I am confident I can replace high sodium foods with lower sodium foods	0.60

Factor 4: priority and concern		
1	Reducing my sodium intake is important to me	0.77
2	My health would improve if I lowered the amount of sodium I eat	0.69
3	I think about sodium in my diet	0.5
4	How concerned are you about the amount of sodium in your diet?	0.68
5	I am concerned about the sodium intake of my family members, relatives and/or friends	0.41

Factor 5: delayed concern		
1	I will likely be concerned about my sodium intake later in my life, but not now	0.50
2	Even if eating too much sodium has negative health effects, I am willing to take the risk	0.49
3	I do not know how to reduce the amount of sodium I eat	0.66
4	It is difficult to understand sodium information on food labels	0.60

Factor 6: sodium-reduction practices		
1	Make your own soups, sauces, and salad dressings	0.64
2	Eat more fresh fruits and vegetables	0.47
3	Eat fewer packaged, ready-to-eat foods	0.64
4	Limit or avoid eating food made in restaurants or cafeterias	0.56
5	To what extent do you personally cook your own meals?	0.42

Abbreviation: EFA, exploratory factor analysis.

To validate the EFA-derived structure, a 6-factor CFA model was tested. The initial model demonstrated moderate fit: CFI = 0.86, TLI = 0.84, RMSEA = 0.067 (95% CI: 0.064, 0.069), SRMR = 0.06. To improve fit, 4 items with factor loadings <0.5 were removed: “Limiting my sodium intake is up to me”; “To what extent do you personally cook your own meals?”; “I am concerned about the sodium intake of my family members, relatives and/or friends”; and “It is difficult to understand sodium information on food labels.” Additionally, 4 theoretically justified interitem correlations were added. These modifications improved model fit: CFI increased to 0.92, TLI to 0.91, RMSEA reduced to 0.056 (95% CI: 0.053, 0.059), SRMR to 0.05, supporting a good model fit (Table 2) (Supplemental Material).

**TABLE 2:** Factor loadings and intraclass correlation of the final CFA 6-factor model for the abbreviated BAIS (BAIS-ab).

	CFA latent factors with related variables	Fully standardized estimate	ICC (95% CI)
Factor 1: nutrition labeling behaviors			
Q1F1	Look at the total amount of sodium (mg) on the Nutrition Facts Table	0.85	0.61 (0.47, 0.72)
Q2F1	Look at the % Daily Value (% DV) on the Nutrition Facts table	0.75	0.59 (0.44, 0.70)
Q3F1	Look for a symbol or a logo on the food package that suggests a product is a healthy choice	0.57	0.57 (0.41, 0.69)
Q4F1	Look for a message or claim on the food package that says a product is “low sodium,” “reduced sodium” or “sodium free”	0.7	0.66 (0.54, 0.76)
Q5F1	Look at the ingredients list for sodium sources	0.78	0.54 (0.38, 0.66)
Q6F1	Read the Nutrition Facts table to determine if a product is high or low sodium	0.8	0.77 (0.68, 0.84)
Q7F1	I am willing to invest time to read food labels to find lower sodium foods	0.8	0.66 (0.53, 0.76)

Factor 2: hedonic influences			
Q1F2	Sodium makes food taste good	0.53	0.65 (0.52, 0.75)
Q2F2	I often have cravings for salty foods	0.87	0.68 (0.55, 0.77)
Q3F2	When I have feelings of stress or sadness, I comfort myself by eating foods that may be high in sodium	0.66	0.55 (0.40, 0.67)
Q4F2	I enjoy eating salty foods or snacks	0.76	0.55 (0.40, 0.67)

Factor 3: perceived control and self-efficacy			
Q1F3	I can control the amount of sodium I consume	0.58	0.34 (0.16, 0.50)
Q2F3	If I wanted to, it would be easy for me to limit the sodium in my diet	0.71	0.48 (0.31, 0.62)
Q3F3	I have willpower to reduce my sodium intake	0.71	0.60 (0.46, 0.71)
Q4F3	I am confident I can identify the sources of sodium in my diet	0.52	0.37 (0.19, 0.53)
Q5F3	I am confident I can replace high sodium foods with lower sodium foods	0.7	0.52 (0.36, 0.65)

Factor 4: priority and concern			
Q1F4	Reducing my sodium intake is important to me	0.86	0.70 (0.58, 0.79)
Q2F4	My health would improve if I lowered the amount of sodium I eat	0.51	0.58 (0.43, 0.70)
Q3F4	I think about sodium in my diet	0.85	0.82 (0.74, 0.87)
Q4F4	How concerned are you about the amount of sodium in your diet?	0.59	0.74 (0.63, 0.81)

Factor 5: delayed concern			
Q1F5	I will likely be concerned about my sodium intake later in my life, but not now	0.71	0.49 (0.32, 0.62)
Q2F5	I do not know how to reduce the amount of sodium I eat	0.55	0.28 (0.09, 0.45)
Q3F5	Even if eating too much sodium has negative health effects, I am willing to take the risk	0.79	0.49 (0.32, 0.62)

Factor 6: sodium-reduction practices			
Q1F6	Make your own soups, sauces, and salad dressings	0.58	0.71 (0.60, 0.80)
Q2F6	Eat more fresh fruits and vegetables	0.58	0.65 (0.51, 0.75)
Q3F6	Eat fewer packaged, ready-to-eat foods	0.71	0.66 (0.53, 0.75)
Q4F6	Limit or avoid eating food made in restaurants or cafeterias	0.59	0.57 (0.42, 0.69)

Abbreviations: CFA, confirmatory factor analysis; CI, confidence interval; ICC, intraclass correlation; QF, question and factor.

#### IRT model assessing binary knowledge variables

IRT analysis of the 11 binary knowledge items indicated good model fit (CFI = 0.93, TLI = 0.91, RMSEA = 0.041, SRMR = 0.04). Discrimination values ranged from 0.17 to 2.35 and difficulty from −2.26 to 8.70. Person-fit was acceptable for most respondents (infit: 97.4%; outfit: 98.0%). Four items with low discrimination and factor loadings <0.4 were removed: perceived sodium impact on health; health conditions associated with sodium; recommended daily sodium intake; and interpretation of sodium levels on a Nutrition Facts table. Model fit improved after their removal (CFI = 0.96, TLI = 0.94, RMSEA = 0.052, SRMR = 0.04), with updated discrimination values from 0.81 to 2.36 and difficulty from −2.62 to 0.15. Person-fit also increased (infit: 99.3%; outfit: 98.0%) [28]. Confirmatory IRT supported the revised model (CFI = 0.96, TLI = 0.94, RMSEA = 0.053, SRMR = 0.04), with all remaining items showing acceptable loadings and parameters. Final person-fit remained high (infit = 0.985; outfit = 0.986; 98.5% and 98.6% within range; refer to Table 3) [28]. See Supplemental Material.

**TABLE 3:** Final confirmatory item response theory 1-factor, 2-parameter logistic model item parameters and AC1 results for the abbreviated BAIS (BAIS-ab).

	Knowledge variables parameters and AC1	*a*	*b*	AC1 (95% CI)
KQ1	This is a warning label that Canadians will soon find on the front of some food packages. What does this label tell you about the food product?	0.81	−2.68	0.81 (0.71, 0.92)
KQ2	To the best of your knowledge, which of the following is the main source of sodium in the Canadian diet?	1.06	−1.57	0.68 (0.53, 0.82)
KQ3	Kosher salt, sea salt and gourmet salts contain less sodium than regular table salt	0.98	0.15	0.56 (0.40, 0.73)
KQ4	My sodium intake is low because I do not add salt to my food	0.87	0.11	0.41 (0.22, 0.59)
KQ5	People with low or normal blood pressure do not need to be concerned with the amount of sodium in their diet	2.36	−0.43	0.48 (0.31, 0.66)
KQ6	Children do not need to limit their sodium intake	2.25	−0.79	0.59 (0.43, 0.75)
KQ7	To the best of your knowledge, how much sodium do you think Canadians eat?	1.58	−1.83	0.82 (0.71, 0.92)

Abbreviations: *a*, discrimination parameter; *b*, difficulty parameter; AC1, Gwet’s first-order agreement coefficient; CI, confidence interval; KQ, knowledge question.

#### Internal consistency and test–retest reliability

Internal consistency estimates (Cronbach’s alpha with 95% CI) for the 6 latent factors were as follows: Nutrition labeling practices = 0.90 (0.89, 0.91), Hedonic influences = 0.79 (0.77, 0.81), Perceived control and self-efficacy = 0.80 (0.78, 0.82), Priority and concern = 0.82 (0.80, 0.83), Delayed concern = 0.73 (0.70, 0.75), and sodium-reduction practices = 0.68 (0.64, 0.71). For the knowledge items, Cronbach’s alpha was 0.64 (0.61, 0.67). Most constructs demonstrated moderate to high internal consistency, suggesting that items within each factor reliably measured their underlying concept [38]. Test–retest reliability of the 6-factor model showed that most ordinal items had moderate reliability, though 6 had poor agreement (Table 2) [36]. In contrast, most knowledge items showed moderate to good agreement, with 2 items demonstrating very good reliability (Table 3) [37].

## Discussion

This study developed and validated a novel, theory-informed instrument to assess cognitive, behavioral, and environmental factors influencing sodium intake among Canadian adults, the BAIS. Using mixed-methods and rigorous psychometric analyses, we identified reliable, distinct 7 constructs that reflect the multilevel determinants of sodium intake. The BAIS demonstrated strong validity and reliability, supporting its use in public health surveillance, intervention evaluation, and policy monitoring.

Existing Canadian and international sodium KAB instruments have made important contributions to the field [[13], [14], [15], [16],[39], [40], [41], [42], [43], [44], [45], [46]]. These tools commonly assessed both declarative and procedural knowledge, including the relationship between salt and sodium, dietary recommendations, population-level intake, dietary sources, and related health outcomes. Attitudinal measures often assessed perceived personal intake relative to recommendations, the importance and benefits of sodium reduction, and levels of concern. Behavioral components frequently included efforts to reduce sodium intake, discretionary salt use, use of nutrition labels, and food choices when eating out. Many instruments also explored the perceived impact of sodium on taste. A Canadian study incorporated constructs from SCT and TPB, yet the survey was administered only in Ontario and targeted a limited age range [14]. The BAIS was informed by these foundational instruments and similarly assessed many of these core constructs. However, it extends beyond previous instruments by incorporating additional cognitive, behavioral, and social factors that were often overlooked, as derived through qualitative research, cognitive interviewing, and the integration of several behavioral theories. The latter includes the incorporation of items related to perceived control, deferred concern, concern regarding sodium intake within one’s social circle, comprehension of front-of-package labeling, and use of sodium substitutes. The BAIS was developed through a theory-driven, multiphase process that incorporated distinctive methods, including quantitative content validity assessment, cognitive interviewing, and psychometric validation, methodological underpinnings that distinguish the BAIS from most existing tools. Its multidimensional structure reflects an evolving understanding of public health nutrition, namely that dietary behavior is shaped by a complex interplay of cognitive, behavioral, and environmental contextual factors. As such, the BAIS offers a more comprehensive and validated instrument for use in population-level research, surveillance, and intervention planning.

Validation analyses strongly supported the internal structure of the instrument. In addition to the knowledge construct, we identified, through EFA, 6 factors with no highly correlated variables (*r* > 0.80), no negative loadings, and low interfactor correlations, indicating item independence and clear conceptual distinctiveness. Moreover, CFA confirmed the dimensionality after removing low-loading items and adding theoretically justified correlations, carefully balancing model fit with theoretical integrity to avoid overfitting. This approach ensured that retained variables meaningfully contributed to their respective constructs by integrating both statistical and conceptual considerations. Furthermore, variability in test–retest reliability may reflect differences in respondent interpretation or sensitivity to change over time. Notably, participant feedback suggested that completing the survey may have prompted reflection or learning, introducing a potential measurement effect.

Several BAIS questions, though supported by strong content and face validity, were not retained in the final factor model (BAIS-ab). Knowledge-related questions that asked to identify the recommended sodium intake and associate health risks with high sodium intake, were excluded due to statistical limitations (e.g., low discrimination relative to other questions) rather than conceptual irrelevance. However, these remain useful in educational and clinical contexts for tracking factual understanding. Similarly, items assessing behaviors including tasting food before adding salt, using discretionary salt or substitutes, rinsing canned vegetables, reading labels, or purchasing low-sodium foods may have been infrequent or context-specific to align with broader behavioral patterns. Cooking and grocery shopping frequencies likely function as background or moderating factors rather than core behavioral drivers, reducing their statistical fit with other items. Although excluded from the final instrument, such questions remain valuable for evaluating program adoption and engagement in sodium-reduction strategies. Questions on perceptions of personal intake, comparisons with average Canadians, concern for others, willingness to pay more, and price sensitivity were conceptually meaningful but may have been situational or heterogeneous to contribute to cohesive latent constructs. Nonetheless, they may support research on risk perception, dietary priorities, and improve risk communication, for example, among younger groups. Social and environmental variables that asked about sodium being high in cultural dishes or the low availability of low sodium options when eating out are important behavioral determinants [17], but their wide variability across contexts limited shared variance in a general population sample. Yet, these domains remain important for understanding structural barriers and cultural influences, particularly in equity focused or culturally tailored interventions. Given that socioeconomic and cultural differences have been reported between English- and French-speaking populations in Canada, it would be interesting to examine whether stratified factor analyses by language yield distinct factor structures [53,54]. Overall, these exclusions reflected limited shared variance, low prevalence, or conceptual uniqueness rather than lack of relevance. These questions may still contribute to program development, hypothesis generation, or targeted interventions, and could be re-examined in subgroup analyses or explored qualitatively to capture their contextual role. Although the factor-derived model improves psychometric coherence and usability, the excluded questions may still provide relevant conceptual insights into sodium-related knowledge, behaviors, and contexts (Supplemental Material).

The BAIS offers a validated tool applicable across multiple settings to support sodium-reduction efforts. In clinical practice, health professionals could use it to identify patients’ knowledge gaps, behavioral challenges, or environmental barriers to lowering sodium intake, thereby informing personalized dietary counseling and goal setting rather than generic advice (e.g., addressing low confidence in label reading or underestimation of sodium in processed foods), particularly among high-risk groups such as those with hypertension or heart disease. In community-based interventions, the BAIS can assess local needs and guide programs that target specific behavioral or cultural drivers of sodium consumption, for example, tailoring nutrition education workshops to identified gaps in knowledge, skills or action, informing targeted SM campaigns by identifying gaps, barriers, and motivators among high-risk populations [19]. In surveillance, it can track sodium-related awareness, behaviors, and beliefs over time and across subgroups, aligning with WHO recommendations [8], and in Canada, could be integrated into the Canadian Community Health Survey. For policy evaluation, the BAIS can assess public responses to initiatives like sodium reformulation or front-of-package labeling, identifying behavioral shifts or persistent barriers such as low perceived risk or limited label comprehension. Where uptake is low despite policy efforts, the BAIS can help determine whether challenges lie in awareness, accessibility, or behavioral resistance. Because Canada adopts upstream sodium-reduction policies, tools like the BAIS are essential not only to measure outcomes but also to understand mechanisms driving change. By clarifying which factors most strongly shape intake, the BAIS can guide evidence-based, context-specific strategies that address both individual and structural determinants of sodium consumption.

Several limitations of this study must be acknowledged. First, although the validation sample was large and mirrored the 2021 Canadian Census to enhance generalizability, it may not have fully captured the perspectives of specific subgroups, including Indigenous communities, newcomers, certain ethnic groups, individuals with limited health literacy, and those with limited internet access or living in remote or rural areas. This may affect the generalizability of findings to these populations, whose unique cultural, socioeconomic, and structural contexts may shape sodium-related KAB in distinct ways. Future research could prioritize oversampling and community-engaged approaches, including partnerships with Indigenous communities and culturally tailored recruitment strategies. Second, reliance on self-reported measures may have introduced social desirability and recall biases. To mitigate these risks, it was emphasized that responses were anonymous and that taking part in the survey was voluntary. We also used validated questions and applied rigorous data quality checks to remove inattentive responses. Third, administering long instruments requires more time, participant effort, and data collection resources, which may pose challenges in resource-limited or time-constrained settings. Future research could explore simplified or modular adaptations to enhance feasibility while maintaining validity. Finally, the initial cognitive interviews for face validity were conducted only in English. Future research could benefit from conducting full rounds of cognitive interviews in both English and French to further improve linguistic and cultural validity. However, the translated French version underwent professional revision and additional review by bilingual participants to ensure clarity and accuracy across both languages.

In conclusion, the BAIS is a rigorously validated, theory-informed instrument that captures multidimensional drivers of sodium intake. It has the potential to generate data to inform the design of targeted interventions and communication strategies, support national surveillance efforts, evaluate public health campaigns, and serve as a reliable outcome measure in intervention studies. More broadly, this study offers a replicable model for public health instrument development, balancing theoretical grounding, user experience, and psychometric rigor.

## Author contributions

The authors’ responsibilities were as follows – JA: supervised the study; JA, WL, RAG: designed the research project; JA, RAG: conducted research, including data collection and cognitive interviews; RAG, MP: analyzed data and performed statistical analysis; RAG: wrote the initial draft article and had primary responsibility for final content; JA, WL, MP: provided critical revisions and feedback on the manuscript; and all authors: read and approved the final manuscript.

## Data availability

Data described in the manuscript, code book, and analytic code will be made available on request pending application to and approval of the corresponding author.

## Funding

This work was supported by the Canadian Institute Health Researchers (PJT187686).

## Conflict of interest

JA reports financial support was provided by Canadian Institutes of Health Research. If there are other authors, they declare that they have no known competing financial interests or personal relationships that could have appeared to influence the work reported in this article.
