# Supplementary material for: Development and Validation of an Instrument to Assess the Cognitive, Behavioral, and Environmental Factors Related to Sodium Intake in Adult Canadians: The Behavioral Assessment Instrument for Dietary Sodium
Source: Curr Dev Nutr. 2025 Oct 30;9(11):107592. doi: 10.1016/j.cdnut.2025.107592 (PMC12670086; doi:10.1016/j.cdnut.2025.107592)
Supplement: Multimedia component 1 [file mmc1.pdf]

Supplementary Material for manuscript '*Development and validation of an instrument to assess the cognitive, behavioural, and environmental factors related to sodium intake in adult Canadians: The Behavioural Assessment Instrument for dietary Sodium (BAIS)*'.

Rola Al Ghali, Michael Prashad, Wendy Lou, and JoAnne Arcand*

Table of contents

Supplementary Material: The Instrument

The Behavioural Assessment Instrument for dietary Sodium (BAIS)-English ………….…………... 2-13

The Behavioural Assessment Instrument for dietary Sodium (BAIS)-French ……………….…. 14-26

The Abbreviated Behavioural Assessment Instrument for dietary Sodium (BAIS-ab)-Factor Model-English ………………………………………………………………………………………….... 27-30

The Abbreviated Behavioural Assessment Instrument for dietary Sodium (BAIS-ab)-Factor Model-French ……………………………………………………………………………….………..…. 31-34

The Behavioural Assessment Instrument for dietary Sodium (BAIS)

1. Using the following scale, how would you rate your overall health? (*Check one*)

Very Poor ⟵⟶				Very Good
1	2	3	4	5

2. Overall, how healthy do you consider your diet to be? (*Check one*)

Very Unhealthy ⟵⟶				Very Healthy
1	2	3	4	5

3. How do you rate your general knowledge related to dietary sodium? *(Check one)*
   a. Very low
   b. Low
   c. Medium
   d. High
   e. Very high

4. To the best of your knowledge, how much do you think sodium affects your overall health? *(Check one)*

Very little ⟵⟶				A great deal	I do not know
(1)	(2)	(3)	(4)	(5)	

5. To the best of your knowledge, which of the following medical conditions are associated or are not associated with high sodium intake? *(Check one per line)*

	Associated	Not associated	I do not know
a. High Blood Pressure	☐	☐	☐
b. Osteoporosis	☐	☐	☐
c. Arthritis	☐	☐	☐
d. Depression	☐	☐	☐
e. Heart Disease	☐	☐	☐
f. Stroke	☐	☐	☐
g. Diabetes	☐	☐	☐
h. Water retention	☐	☐	☐
i. Weight gain	☐	☐	☐
j. Abdominal Bloating	☐	☐	☐
k. Anxiety	☐	☐	☐
l. Kidney disease	☐	☐	☐

6. To the best of your knowledge, what is the maximum amount of sodium that adults should have in one day? *(Check one)*

a. 900 mg	☐
b. 1200 mg	☐
c. 1500 mg	☐
d. 2000 mg	☐
e. 2300 mg	☐
f. 2800 mg	☐
g. 3400 mg	☐
h. I do not know	☐

7. This is a Nutrition Facts table for a box of crackers. If we were to ask you to judge how much sodium is in this product, would you say it is low, medium, or high sodium? *(Check one) - Subjects to be randomly assigned one of the following low, medium, or high Nutrition Facts tables*

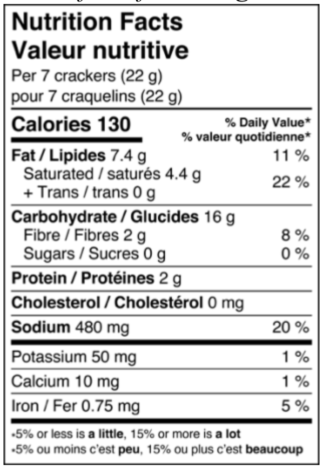

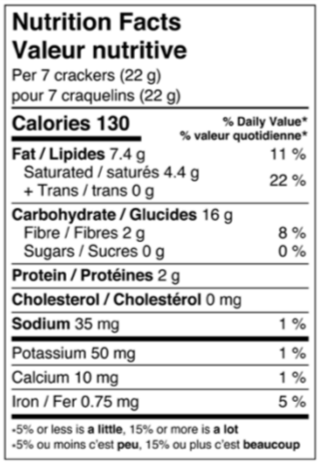

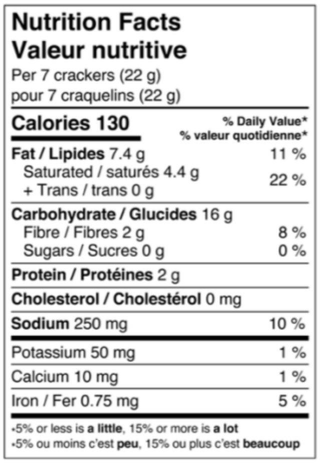

a. Low	a. Low	a. Low
b. Medium	b. Medium	b. Medium
c. High	c. High	c. High
d. I do not know	d. I do not know	d. I do not know

8. This is a warning label that Canadians will soon find on the front of some food packages. What does this label tell you about the food product? *(Check one)*

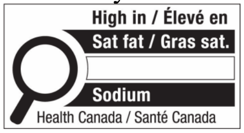

a. The food is *low* in sodium and saturated fat
b. The food is *high* in sodium and saturated fat
c. The food is *high* in saturated fat and *low* in sodium
d. The food *does not contain* sodium and saturated fat
e. I do not know

9. To the best of your knowledge, which of the following is the main source of sodium in the Canadian diet? *(Check one)*
   a. Sodium added during cooking
   b. Sodium added at the table
   c. Sodium added to processed and prepared foods
   d. Sodium that is naturally present in food
   e. I do not know

10. Please tell us to what extent you agree or disagree with the following statements. *(Check one per line)*

	Strongly Disagree ↔ Strongly Agree				
	1	2	3	4	5
a. Kosher salt, sea salt and gourmet salts contain less sodium than regular table salt					
b. My sodium intake is low because I do not add salt to my food					
c. People with low or normal blood pressure do not need to be concerned with the amount of sodium in their diet					
d. Children do not need to limit their sodium intake					

11. To the best of your knowledge, how much sodium do you think Canadians eat? *(Check one)*
   a. Far too much
   b. Too much
   c. The right amount
   d. Too little
   e. Far too little
   f. I do not know

12. Which of the following best describes your approach towards sodium? *(Check one)*
   a. I am not limiting my sodium intake. (*Skip to Q14)*
   b. I am interested in limiting my sodium intake, but have not yet started. (*Go to Q13 then 16*)
   c. I have tried limiting my sodium intake in the past, but not anymore. (*Go to Q13 then 16*)
   d. I am currently trying to limit my sodium intake. (*Go to Q13 then 16*)

Only asked to those LIMITING sodium, based on Q12: "I am interested in limiting my sodium intake", "I have tried limiting my sodium intake but not anymore", "I am currently trying to limit my sodium intake". Participants who are answering "LIMITING" will answer Q 13 then move to Q16.

13. Please tell us the extent to which you agree or disagree with the following statements as reasons for limiting your sodium intake. *(Check one per line)*

	Strongly Disagree ↔				Strongly Agree	Not Applicable
	1	2	3	4	5	
a. To reduce my chances of developing a health condition in the future						
b. To manage a current health condition						
c. Because my health care provider has recommended it						
d. Because I want to reduce or stop my blood pressure medication						
e. Because people close to me, such as family members, relatives and/or friends, are limiting their sodium intake						

Participants who are answering "NOT LIMITING" will answer Q14 and Q15 then move to Q16. Only asked to those NOT LIMITING, based on Q12: "I do not need to limit my sodium intake" and "I am not interested in limiting my sodium intake".

14. Please tell us the extent to which you agree or disagree with the following statements as reasons for not limiting your sodium intake. *(Check one per line)*

	Strongly Disagree ↔				Strongly Agree	Not Applicable
	1	2	3	4	5	
a. I do not have a medical condition to control with a lower sodium diet						
b. Sodium is not bad for me						
c. There is conflicting information about whether I should or should not reduce my sodium						
d. I have a normal or low blood pressure						
e. I do not need to limit my sodium since I am on blood pressure medication						
f. My health care provider has not recommended it						
g. People close to me, such as family members, relatives and/or friends, are not limiting their sodium intake						

Only asked to those NOT LIMITING, based on Q12: "I do not need to limit my sodium intake" and "I am not interested in limiting my sodium intake".

15. Please help us understand what might motivate you if you were to limit your sodium intake? *(Check one per line)*

	Strongly disagree ↔				Strongly Agree	Not Applicable
	1	2	3	4	5	
a. If people close to me, such as family members, relatives and/or friends, encouraged me to lower my sodium intake						
b. If people close to me, such as family members, relatives and/or friends, were limiting their sodium intake						
c. If limiting my sodium intake would help me reduce or stop my blood pressure medication						
d. If limiting my sodium intake would reduce my chances of developing a health condition						
e. If limiting my sodium intake would help me manage a health condition						
f. If my health care provider recommended it						

16. Over the past month, can you think of an occasion when you decided to avoid purchasing or consuming a particular food because you thought it was too high in sodium? *(Check one)*
    a. Yes
    b. No
    c. I do not know/remember

17. Have you heard of low-sodium salts, also called "salt alternatives" or "salt substitutes" (e.g., potassium chloride salt)? *(Check one)*
    a. Yes
    b. No

18. Below we have listed some of the common ways to lower the amount of sodium in your diet. In the past month, to what extent have you personally done any of the following, regardless of whether or not you are trying to limit your sodium? (*Check one per line*)

	Never do this ↔				Always do this	Not Applicable
	1	2	3	4	5	
a. Avoid adding salt during cooking						
b. Avoid adding salt at the table						
c. Make your own soups, sauces, and salad dressings						
d. Eat more fresh fruits and vegetables						
e. Eat fewer packaged, ready-to-eat foods						
f. Taste your food before adding salt						
g. Use spices, herbs and/or seasonings instead of salt during cooking						
h. Drain and rinse canned vegetables and beans/legumes before use						
i. Limit or avoid eating food made in restaurants or cafeterias						
j. At restaurants, ask to have your meal prepared without salt						
k. At restaurants, ask for dressings and sauces on the side						
l. Read the Nutrition Facts table to determine if a product is high or low sodium *(answers 3, 4, or 5 go to Q19)*						
m. Buy foods labelled as “low sodium” or “reduced sodium” or “sodium free”, when available						
n. Use low-sodium salts, also called “salt alternatives” or “salt substitutes” (e.g., potassium chloride salt) instead of regular salt						

19. Imagine you are comparing sodium content between two similar products. How do you typically decide which one to buy? *(Check one)*
    a. I purchase the one lower in sodium
    b. I purchase the less expensive option
    c. I purchase the option I think is healthier overall
    d. I purchase the one I think will taste best
    e. I purchase the brand I/my family prefers
    f. I purchase the one higher in sodium
    g. I do not compare nutrition labels between products

20. This is a warning label that Canadians will soon see on the front of some food packages. How likely would you be to eat a product that has this label displayed on the front of its package? *(Check one)- Subjects to be randomly assigned one of the following FOPs: high in fat and sodium (A), high in fat, sodium, and sugar (B), and high in sodium (C).*

A

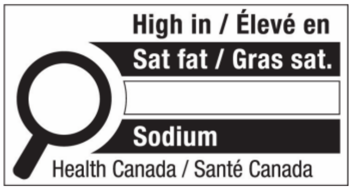

a. Very unlikely
b. Unlikely
c. Neutral
d. Likely
e. Very likely

B

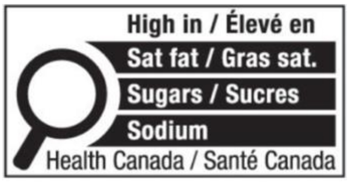

a. Very unlikely
b. Unlikely
c. Neutral
d. Likely
e. Very likely

C

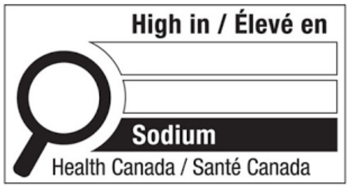

a. Very unlikely
b. Unlikely
c. Neutral
d. Likely
e. Very likely

21. When there are many products to choose from, we are interested to know how you would find the lowest sodium choice. To what extent do you/would you do the following in order to find the lowest sodium product? *(Check one per line)*

	Never do this ↔				Always do this
	1	2	3	4	5
a. Look at the total amount of sodium (mg) on the Nutrition Facts table					
b. Look at the % Daily Value (% DV) on the Nutrition Facts table					
c. Look for a symbol or a logo on the food package that suggests a product is a healthy choice					
d. Look for a message or claim on the food package that says a product is “low sodium”, “reduced sodium” or “sodium free”					
e. Look at the ingredients list for sodium sources					

22. Which of the following best reflects your use of low- sodium salts at home, also called “salt alternatives” or “salt substitutes” (e.g., potassium chloride salt)? *(Check one)*
    a. I currently use low sodium salts
    b. In the past I have used low sodium salts, but not anymore
    c. I have tried low sodium salts, but I do not use them at home
    d. I have not tried or used low sodium salts before
    e. I do not remember

23. Please comment on your use, or lack of use, of low sodium salts at home.
    Open ended question: ____________________

24. How much sodium do you think you consume? *(Check one)*
    a. Far too much
    b. Too much
    c. The right amount
    d. Too little
    e. Far too little
    f. I do not know

25. How do you think your <u>own</u> intake of sodium <u>compares</u> with the average Canadian adult? (*Check one*)
    a. Much lower
    b. Somewhat lower
    c. About the same
    d. Somewhat higher
    e. Much higher
    f. I do not know

26. Considering your diet, choose the top source for most of your sodium. *(Check one)*
    a. Packaged food (e.g., canned food, frozen meal)
    b. Prepared food (e.g., potato salad from the grocery deli, salad bar items)
    c. Salt added while eating
    d. Salt added during cooking
    e. Restaurant foods
    f. Other, please specify:________
    g. I do not know

27. Please tell us the extent to which you agree or disagree with the following statements. *(Check one per line)*

	Strongly Disagree ↔				Strongly Agree
	1	2	3	4	5
a. I think about sodium in my diet					
b. Reducing my sodium intake is important to me					
c. My health would improve if I lowered the amount of sodium I eat					

28. If you found out you were eating too much sodium, how likely would you be to take steps to lower your intake? *(Check one)*

Very unlikely	↔			Very Likely
1	2	3	4	5
☐	☐	☐	☐	☐

29. How concerned are you about the amount of sodium in your diet? *(Check one)*
    a. Extremely concerned
    b. Very concerned
    c. Somewhat concerned
    d. Not very concerned
    e. Not at all concerned

30. Please tell us to what extent you agree or disagree with the following statements. *(Check one per line)*

	Strongly Disagree ↔				Strongly Agree
	1	2	3	4	5
a. I will likely be concerned about my sodium intake later in my life, but not now					
b. I am concerned about the sodium intake of my family members, relatives and/or friends					
c. Even if eating too much sodium has negative health effects, I am willing to take the risk					

31. Please tell us the extent to which you agree or disagree with the following statements. *(Check one per line)*

	Strongly Disagree ↔				Strongly Agree	Not Applicable
	1	2	3	4	5	
a. Sodium makes food taste good						
b. In general, lower sodium food products do not taste as good, compared to regular products						
c. I often have cravings for salty foods						
d. When I have feelings of stress or sadness, I comfort myself by eating foods that may be high in sodium						
e. I enjoy eating salty foods or snacks						
f. I do not like foods that taste salty						

32. Please tell us the extent to which you agree or disagree with the following statements, regardless of whether or not you are trying to limit your sodium intake. *(Check one per line)*

	Strongly Disagree ↔				Strongly Agree	Not Applicable
	1	2	3	4	5	
a. Within reason, I am willing to pay more for lower sodium foods						
b. I am willing to invest time to read food labels to find lower sodium foods						
c. I am willing to sacrifice taste in order to limit my sodium intake						
d. The price difference between low-sodium and regular foods is too high for me						
e. I do not always have time to prepare lower sodium meals from scratch						

33. Several social factors may affect lowering the amount of sodium in your diet. Please tell us the extent to which you agree or disagree with the following statements, regardless of whether or not you are trying to limit your sodium intake. *(Check one per line)*

	Strongly Disagree ↔				Strongly Agree	Not Applicable
	1	2	3	4	5	
a. A lack of support from my family members, relatives and/or friends makes reducing sodium difficult						
b. Using higher sodium food products or ingredients is part of my cultural dishes						
c. Reducing dietary sodium is difficult because no one in my household is limiting their sodium intake						
d. When I am dining with others, I do not pay attention to the sodium content of the food choices						
e. People close to me such as family members, relatives and/or friends have encouraged me to follow a lower sodium diet						

34. To what extent do you agree or disagree that: "People important to me want me to limit my sodium intake"? *(Check one)*

Strongly Disagree ↔				Strongly Agree	I do not know
1	2	3	4	5	

35. Several environmental factors may affect lowering the amount of sodium in your diet. Please tell us the extent to which you agree or disagree with the following statements, regardless of whether or not you are trying to limit your sodium intake. *(Check one per line)*

	Strongly Disagree ↔				Strongly Agree	Not Applicable
	1	2	3	4	5	
a. When shopping for groceries, I find that lower sodium options are not available or only in limited variety						
b. It is difficult to find low sodium options on online food retail and/or delivery platforms						
c. When eating at fast food restaurants, I find that lower sodium options are not available or only in limited variety						
d. When eating at sit-down restaurants, I find that lower sodium options are not available or only in limited variety						

36. Please tell us the extent to which you agree or disagree with the following statements regarding your control over your sodium intake, regardless of whether or not you are trying to limit your sodium intake. *(Check one per line)*

	Strongly Disagree ↔				Strongly Agree
	1	2	3	4	5
a. I can control the amount of sodium I consume					
b. Limiting my sodium intake is up to me					
c. If I wanted to, it would be easy for me to limit the sodium in my diet					
d. I have willpower to reduce my sodium intake					

37. Please tell us the extent to which you agree or disagree with the following statements, regardless of whether or not you are trying to limit your sodium intake. *(Check one per line)*

	Strongly Disagree ↔				Strongly Agree	Not Applicable
	1	2	3	4	5	
a. I do not know how to reduce the amount of sodium I eat						
b. It is difficult to understand sodium information on food labels						
c. I am confident I can prepare my food from scratch at home						
d. I am confident I can identify the sources of sodium in my diet						
e. I am confident I can replace high sodium foods with lower sodium foods						

38. How often do you personally do the grocery shopping in your household? *(Check one)*
    a. Never
    b. Rarely
    c. Sometimes
    d. Very often
    e. Always
    f. Prefer not to answer

39. To what extent do you personally cook your own meals? *(Check one)*
    a. I cook all my meals
    b. I cook the majority of my meals
    c. I cook half of my meals
    d. I cook some of my meals
    e. I do not cook my meals
    f. Prefer not to answer

40. Please indicate how much you agree or disagree with the following statement: "I choose food products based on experience or knowledge and not on food labels". *(Check one)*

Strongly Disagree ↔				Strongly Agree
1	2	3	4	5

41. The following question asks about your eating behaviours in the past month. When answering, please consider all meals such as, breakfast, lunch, dinner/supper, and snacks. *(Check one per line)*

	Never	1-3 times/ month	1-3 times/ week	4-6 times/ week	7 or more times/ week
I eat food from restaurants. This includes dine-in restaurants, take-out, delivery or fast food.					

Instrument D'Évaluation Comportementale pour le Sodium alimentaire (BAIS)

1. En utilisant l'échelle suivante, comment évalueriez-vous votre santé globale? (*Sélectionnez une seule réponse*)

Très mauvaise	⟷			Très bonne
1	2	3	4	5

2. De manière générale, dans quelle mesure considérez-vous que votre alimentation est saine? (*Sélectionnez une seule réponse*)

Très peu saine	⟷			Très saine
1	2	3	4	5

3. Comment évaluez-vous vos connaissances générales en ce qui concerne le sodium alimentaire? *(Sélectionnez une seule réponse)*
   a. Très faibles
   b. Faibles
   c. Moyennes
   d. Bonnes
   e. Très bonnes

4. À votre connaissance, dans quelle mesure le sodium affecte-t-il votre santé globale? *(Sélectionnez une seule réponse)*

Très peu	⟷			Beaucoup	Je ne sais pas
(1)	(2)	(3)	(4)	(5)	

5. À votre connaissance, lesquelles des affections ou maladies suivantes sont associées ou non associées à un apport élevé de sodium? *Sélectionnez une réponse dans chaque ligne.*

	Associé	Non associé	Je ne sais pas
a. Haute pression sanguine			
b. Ostéoporose			
c. Arthrite			
d. Dépression			
e. Maladie cardiaque			
f. Accident cérébrovasculaire			
g. Diabète			
h. Rétention d'eau			
i. Prise de poids			
j. Ballonnement abdominal			
k. Anxiété			
l. Maladie rénale			

6. À votre connaissance, quelle est la quantité maximale de sodium que les adultes devraient consommer en une journée? *(Sélectionnez une seule réponse)*

a. 900 mg	
b. 1200 mg	
c. 1500 mg	
d. 2000 mg	
e. 2300 mg	
f. 2800 mg	
g. 3400 mg	
h. Je ne sais pas	

7. "Il existe un certain nombre de marques de craquelins dont la teneur en sodium varie de faible à élevée. Sur le prochain écran, nous allons vous montrer un tableau de la valeur nutritive d'une de ces boîtes de craquelins. » Voici un tableau de la valeur nutritive d'une boîte de craquelins. Si nous vous demandions d'estimer la quantité de sodium contenue dans ce produit, diriez-vous que sa teneur en sodium est faible, moyenne ou élevée? *(Sélectionnez une seule réponse)*

**Nutrition Facts** **Valeur nutritive**	
Per 7 crackers (22 g) pour 7 craquelins (22 g)	
**Calories 130**	**% Daily Value*** **% valeur quotidienne***
**Fat / Lipides** 7.4 g	11 %
Saturated / saturés 4.4 g + Trans / trans 0 g	22 %
**Carbohydrate / Glucides** 16 g	
Fibre / Fibres 2 g	8 %
Sugars / Sucres 0 g	0 %
**Protein / Protéines** 2 g	
**Cholesterol / Cholestérol** 0 mg	
**Sodium** 480 mg	20 %
Potassium 50 mg	1 %
Calcium 10 mg	1 %
Iron / Fer 0.75 mg	5 %
*5% or less is **a little**, 15% or more is **a lot** *5% ou moins c'est **peu**, 15% ou plus c'est **beaucoup**	

a. Faible
b. Moyenne
c. Élevée
d. Je ne sais pas

**Nutrition Facts** **Valeur nutritive**	
Per 7 crackers (22 g) pour 7 craquelins (22 g)	
**Calories 130**	**% Daily Value*** **% valeur quotidienne***
**Fat / Lipides** 7.4 g	11 %
Saturated / saturés 4.4 g + Trans / trans 0 g	22 %
**Carbohydrate / Glucides** 16 g	
Fibre / Fibres 2 g	8 %
Sugars / Sucres 0 g	0 %
**Protein / Protéines** 2 g	
**Cholesterol / Cholestérol** 0 mg	
**Sodium** 35 mg	1 %
Potassium 50 mg	1 %
Calcium 10 mg	1 %
Iron / Fer 0.75 mg	5 %
*5% or less is **a little**, 15% or more is **a lot** *5% ou moins c'est **peu**, 15% ou plus c'est **beaucoup**	

a. Faible
b. Moyenne
c. Élevée
d. Je ne sais pas

**Nutrition Facts** **Valeur nutritive**	
Per 7 crackers (22 g) pour 7 craquelins (22 g)	
**Calories 130**	**% Daily Value*** **% valeur quotidienne***
**Fat / Lipides** 7.4 g	11 %
Saturated / saturés 4.4 g + Trans / trans 0 g	22 %
**Carbohydrate / Glucides** 16 g	
Fibre / Fibres 2 g	8 %
Sugars / Sucres 0 g	0 %
**Protein / Protéines** 2 g	
**Cholesterol / Cholestérol** 0 mg	
**Sodium** 250 mg	10 %
Potassium 50 mg	1 %
Calcium 10 mg	1 %
Iron / Fer 0.75 mg	5 %
*5% or less is **a little**, 15% or more is **a lot** *5% ou moins c'est **peu**, 15% ou plus c'est **beaucoup**	

a. Faible
b. Moyenne
c. Élevée
d. Je ne sais pas

8. Ceci est une étiquette de mise en garde que les Canadien·ne·s trouveront bientôt sur le devant de certains emballages de produits alimentaires. Que vous apprend cette étiquette sur le produit alimentaire? *(Sélectionnez une seule réponse)*

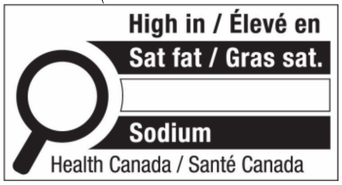

   a. L'aliment a une *faible* teneur en sodium et en gras saturés
   b. L'aliment a une teneur *élevée* en sodium et en gras saturés
   c. L'aliment a une teneur *élevée* en gras saturés et faible en sodium
   d. L'aliment *ne contient pas* de sodium ni de gras saturés
   e. Je ne sais pas

9. À votre connaissance, laquelle des sources suivantes est la principale source de sodium dans l'alimentation des Canadiens? *(Sélectionnez une seule réponse)*
   a. Sodium ajouté pendant la cuisson
   b. Sodium ajouté à table
   c. Sodium ajouté aux aliments transformés et préparés
   d. Sodium naturellement présent dans les aliments
   e. Je ne sais pas

10. Veuillez indiquer dans quelle mesure vous êtes d'accord ou en désaccord avec les affirmations suivantes. *(Sélectionnez une seule réponse chaque ligne)*

	Tout à fait en désaccord ↔				Tout à fait d'accord
	1	2	3	4	5
a. Le sel casher, le sel de mer et les sels de spécialité contiennent moins de sodium que le sel de table ordinaire					
b. Mon apport en sodium est faible parce que je n'ajoute pas de sel à mes aliments					
c. Les personnes dont la tension artérielle est basse ou normale n'ont pas à se préoccuper de la quantité de sodium dans leur alimentation					
d. Les enfants n'ont pas besoin de limiter leur consommation de sodium					

11. À votre avis, quelle quantité de sodium est consommée par les Canadien·ne·s? *(Sélectionnez une seule réponse)*
   a. Beaucoup trop
   b. Trop
   c. Juste la quantité qu'il faut
   d. Trop peu
   e. Beaucoup trop peu
   f. Je ne sais pas

12. Lequel des comportements suivants décrit le mieux votre propre approche à l'égard du sodium? *(Sélectionnez une seule réponse)*
  a. Je ne limite pas ma consommation de sodium.
  b. Je souhaite limiter ma consommation de sodium, mais je n'ai pas encore commencé à le faire.
  c. J'ai essayé de limiter ma consommation de sodium dans le passé, mais je ne le fais plus maintenant.
  d. J'essaie actuellement de limiter ma consommation de sodium.

13. Veuillez indiquer dans quelle mesure vous êtes d'accord ou en désaccord pour dire que <u>les raisons suivantes vous ont incité·e à limiter votre consommation de sodium</u>. *Sélectionnez une seule réponse chaque ligne)*

	Tout à fait en désaccord ↔				Tout à fait d'accord	Ne s'applique pas
	1	2	3	4	5	
a. Pour réduire mon risque de développer un problème de santé à l'avenir						
b. Pour gérer un problème de santé actuel						
c. Parce que mon prestataire de soins de santé me l'a recommandé						
d. Parce que je veux réduire ou arrêter de prendre mon médicament contre la tension artérielle						
e. Parce que mes proches, tels que ma famille immédiate, les autres membres de ma famille et/ou des amis limitent leur consommation de sodium						

14. Veuillez indiquer dans quelle mesure vous êtes d'accord ou en désaccord pour dire que <u>les raisons suivantes vous incitent à ne pas limiter votre consommation de sodium</u>. *(Sélectionnez une seule réponse chaque ligne)*

	Tout à fait en désaccord ↔				Tout à fait d'accord	Ne s'applique pas
	1	2	3	4	5	
a. Je ne souffre pas d'un problème de santé qui doit être contrôlé avec une alimentation pauvre en sodium						
b. Le sodium n'est pas mauvais pour moi						
c. Les informations sont contradictoires sur la question de savoir si je dois ou non réduire ma consommation de sodium						
d. J'ai une tension artérielle normale ou basse						
e. Je n'ai pas besoin de limiter ma consommation de sodium, car je prends des médicaments contre la tension artérielle						
f. Mon prestataire de soins de santé ne me l'a pas recommandé						
g. Mes proches, tels que les membres de ma famille, mes parents et/ou mes ami·e·s, ne limitent pas leur consommation de sodium						

15. Veuillez nous aider à comprendre ce qui pourrait vous motiver à limiter votre consommation de sodium. *(Sélectionnez une seule réponse chaque ligne)*

	Tout à fait en désaccord ↔ Tout à fait d'accord					Ne s'applique pas
	1	2	3	4	5	
a. Si mes proches, tels que ma famille immédiate, les autres membres de ma famille et/ou des ami·e·s, m'encouragent à réduire ma consommation de sodium						
b. Si mes proches, tels que ma famille immédiate, les autres membres de ma famille et/ou des ami·e·s, limitent leur consommation de sodium						
c. Si le fait de limiter ma consommation de sodium m'aide à réduire ou à arrêter de prendre mon médicament contre la tension artérielle						
d. Si le fait de limiter ma consommation de sodium aide à réduire mon risque de développer un problème de santé						
e. Si le fait de limiter ma consommation de sodium m'aide à gérer un problème de santé						
f. Si mon prestataire de soins de santé me le recommande						

16. Au cours du dernier mois, vous souvenez-vous d'une occasion où vous avez décidé de ne pas acheter ou consommer un aliment spécifique parce que vous avez pensé que sa teneur en sodium était trop élevée? *(Sélectionnez une seule réponse)*
a. Oui
b. Non
c. Je ne sais pas/je ne me souviens pas

17. Avez-vous déjà entendu parler de sels à faible teneur en sodium, également appelés « solutions de rechange au sel » ou « succédanés du sel » (p. ex. sel de chlorure de potassium)? *(Sélectionnez une seule réponse)*
a. Oui
b. Non

18. Vous trouverez ci-dessous une liste des moyens les plus courants pour réduire la quantité de sodium dans votre alimentation. Au cours du dernier mois, dans quelle mesure avez-vous personnellement fait l'une des choses suivantes, que vous essayiez ou non de limiter votre consommation de sodium? (*Sélectionnez une seule réponse chaque ligne*)

	Je ne fais jamais ceci ↔ Je fais toujours ceci					Ne s'applique pas
	1	2	3	4	5	
a. Éviter d'ajouter du sel pendant la cuisson						
b. Éviter d'ajouter du sel à table						
c. Préparer vous-même vos soupes, sauces et vinaigrettes						
d. Manger plus de fruits et de légumes frais						
e. Manger moins d'aliments emballés et prêts à consommer						
f. Goûter vos aliments avant d'ajouter du sel						
g. Utiliser des épices, des herbes fines et/ou des assaisonnements plutôt que du sel pendant la cuisson						
h. Drainer et rincer les légumes et les haricots/légumineuses en conserve avant de les utiliser						
i. Limiter ou éviter de manger des aliments préparés dans les restaurants ou cafétérias						
j. Au restaurant, demander que votre plat soit préparé sans sel						
k. Au restaurant, demander que les vinaigrettes et les sauces vous soient servies à part						
l. Lire le tableau de la valeur nutritive pour déterminer si un produit a une teneur en sodium faible ou élevée						
m. Acheter des aliments étiquetés « à faible teneur en sodium » ou « à teneur réduite en sodium » ou « sans sodium », lorsqu'ils sont disponibles						
n. Utiliser des sels à faible teneur en sodium, également appelés « succédanés du sel » ou « substituts de sel » (p. ex. le sel de chlorure de potassium) au lieu du sel ordinaire						

19. Imaginez que vous compariez la teneur en sodium de deux produits similaires. Comment décidez-vous généralement de celui que vous allez acheter? *(Sélectionnez une seule réponse)*
a. J'achète celui qui contient le moins de sodium
b. J'achète l'option la moins chère
c. J'achète l'option qui me semble la plus saine globalement
d. J'achète celui qui me semble avoir meilleur goût
e. J'achète la marque que je préfère ou que ma famille préfère
f. J'achète celui qui a une teneur en sodium plus élevée
g. Je ne compare pas les étiquettes nutritionnelles des différents produits

20. Ceci est une étiquette de mise en garde que les Canadien·ne·s trouveront bientôt sur certains emballages de produits alimentaires. Quelle est la probabilité que vous mangiez un produit qui porte cette étiquette sur le devant de son emballage? *(Sélectionnez une seule réponse)*

A

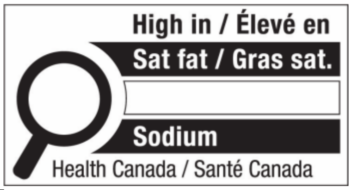

a. Très improbable
b. Improbable
c. Neutre
d. Probable
e. Très probable

B

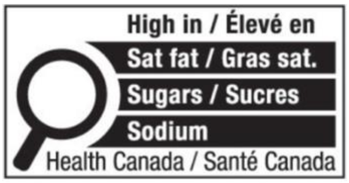

a. Très improbable
b. Improbable
c. Neutre
d. Probable
e. Très probable

C

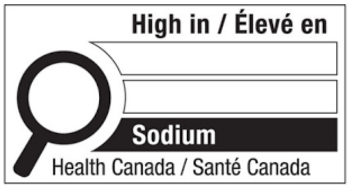

a. Très improbable
b. Improbable
c. Neutre
d. Probable
e. Très probable

21. Lorsque vous avez le choix entre de nombreux produits, nous aimerions savoir comment vous vous y prenez pour trouver le choix qui contient le moins de sodium. Dans quelle mesure faites-vous ou feriez-vous ce qui suit afin de trouver le produit qui a la plus faible teneur en sodium? *(Sélectionnez une seule réponse chaque ligne)*

	Je ne fais jamais ceci ↔				Je fais toujours ceci
	1	2	3	4	5
a. Vérifier la teneur totale en sodium (mg) dans le tableau de la valeur nutritive					
b. Vérifier le % de valeur quotidienne (% VQ) dans le tableau de la valeur nutritive					
c. Regarder sur l'emballage du produit alimentaire s'il y a un symbole ou un logo qui suggère que le produit est un choix sain					
d. Regarder sur l'emballage du produit alimentaire s'iI y a un message ou une allégation qui indique que le produit est « faible en sodium », « à teneur réduite en sodium » ou « sans sodium »					
e. Vérifier les sources de sodium dans la liste des ingrédients					

22. Lequel des énoncés suivants reflète le mieux votre utilisation des sels à faible teneur en sodium à la maison, également appelés « solutions de rechange au sel » ou « succédanés du sel » (p. ex. sel de chlorure de potassium)? *(Sélectionnez une seule réponse)*

a. J'utilise actuellement des sels à faible teneur en sodium
b. J'ai déjà utilisé des sels à faible teneur en sodium dans le passé, mais plus maintenant
c. J'ai déjà essayé les sels à faible teneur en sodium, mais je ne les utilise pas à la maison
d. Je n'ai jamais essayé ni utilisé les sels à faible teneur en sodium
e. Je ne me souviens pas

23. Veuillez expliquer pourquoi vous utilisez ou n'utilisez pas du sel à faible teneur en sodium à la maison.

Question ouverte :________

24. À votre avis, quelle quantité de sodium consommez-vous? (Sélectionnez une seule réponse)
a. Beaucoup trop
b. Trop
c. Juste la quantité qu'il faut
d. Trop peu
e. Beaucoup trop peu
f. Je ne sais pas

25. À votre avis, comment se compare votre consommation personnelle de sodium à celle de l'adulte canadien moyen? (*Sélectionnez une seule réponse*)
a. Beaucoup plus faible
b. Un peu plus faible
c. À peu près la même
d. Un peu plus élevée
e. Beaucoup plus élevée
f. Je ne sais pas

26. En considérant votre alimentation, veuillez choisir la source principale de la majeure partie de votre apport en sodium *(Sélectionnez une seule réponse)*
a. Aliments emballés (p. ex. conserves, repas surgelés)
b. Aliments préparés (p. ex. salade de pommes de terre prête-à-manger de l'épicerie, options dans les bars à salade)
c. Sel ajouté pendant le repas
d. Sel ajouté pendant la cuisson
e. Aliments au restaurant
f. Autre (veuillez préciser) :
g. Je ne sais pas

27. Veuillez indiquer dans quelle mesure vous êtes d'accord ou en désaccord avec les affirmations suivantes. *(Sélectionnez une réponse dans chaque ligne.)*

	Tout à fait en désaccord	↔			Tout à fait d'accord
	1	2	3	4	5
a. Je tiens compte du sodium dans mon alimentation					
b. Il est important pour moi de réduire ma consommation de sodium					
c. Ma santé s'améliorerait si je réduisais la quantité de sodium que je consomme					

28. Si vous appreniez que vous consommez trop de sodium, quelle serait la probabilité que vous preniez des mesures pour réduire votre consommation? *(Sélectionnez une seule réponse)*

Très improbable	←→			Très probable
1	2	3	4	5

29. Dans quelle mesure êtes-vous préoccupé·e par la quantité de sodium dans votre alimentation? *(Sélectionnez une seule réponse)*
a. Extrêmement préoccupé·e
b. Très préoccupé·e
c. Assez préoccupé·e
d. Peu préoccupé·e
e. Pas du tout préoccupé·e

30. Veuillez indiquer dans quelle mesure vous êtes d'accord ou en désaccord avec les affirmations suivantes. *(Sélectionnez une seule réponse chaque ligne)*

	Tout à fait en désaccord	←→			Tout à fait d'accord
	1	2	3	4	5
a. Je m'inquièterais probablement de ma consommation de sodium plus tard dans ma vie, mais pas maintenant					
b. Je m'inquiète de la consommation de sodium de ma famille immédiate, des autres membres de ma famille et/ou de mes ami·e·s					
c. Même si le fait de consommer trop de sodium a des effets négatifs sur ma santé, je suis prêt·e à en prendre le risque					

31. Veuillez indiquer dans quelle mesure vous êtes d'accord ou en désaccord avec les affirmations suivantes. *(Sélectionnez une réponse dans chaque ligne)*

	Tout à fait en désaccord	←→			Tout à fait d'accord	Ne s'applique pas
	1	2	3	4	5	
a. Le sodium donne du goût aux aliments						
b. En général, les produits alimentaires à teneur réduite en sodium n'ont pas le même goût que les produits ordinaires						
c. J'ai souvent des envies d'aliments salés						
d. Lorsque je ressens du stress ou de la tristesse, je me réconforte en mangeant des aliments qui peuvent être riches en sodium						
e. J'aime manger des collations ou des aliments salés						
f. Je n'aime pas les aliments au goût salé						

32. Veuillez indiquer dans quelle mesure vous êtes d’accord ou en désaccord avec les affirmations suivantes, que vous essayiez ou non de limiter votre consommation de sodium. *(Sélectionnez une réponse dans chaque ligne.)*

	Tout à fait en désaccord ◄──► Tout à fait d'accord					Ne s'applique pas
	1	2	3	4	5	
a. Dans la limite du raisonnable, je suis prêt·e à payer plus cher pour acheter des aliments à teneur réduite en sodium						
b. Je suis prêt·e à consacrer du temps à la lecture des étiquettes des produits alimentaires pour trouver des aliments à faible teneur en sodium						
c. Je suis prêt·e à sacrifier le goût pour limiter ma consommation de sodium						
d. La différence de prix entre les aliments à faible teneur en sodium et les aliments ordinaires est trop importante pour moi						
e. Je n'ai pas toujours le temps de préparer des repas à faible teneur en sodium à partir de zéro						

33. Plusieurs facteurs sociaux peuvent influer sur la réduction de la quantité de sodium dans votre alimentation. Veuillez indiquer dans quelle mesure vous êtes d’accord ou en désaccord avec les affirmations suivantes, que vous essayiez ou non de limiter votre consommation de sodium. *(Sélectionnez une réponse dans chaque ligne.)*

	Tout à fait en désaccord ◄──► Tout à fait d'accord					Ne s'applique pas
	1	2	3	4	5	
a. Un manque de soutien de la part de ma famille immédiate, des autres membres de ma famille et/ou de mes ami·e·s rend plus difficile pour moi de réduire le sodium que je consomme						
b. L'utilisation de produits ou d'ingrédients alimentaires à forte teneur en sodium fait partie de mes plats culturels						
c. Il est difficile de réduire le sodium alimentaire, car personne dans mon foyer ne limite sa consommation de sodium						
d. Lorsque je partage un repas avec d'autres personnes, je ne fais pas attention à la teneur en sodium des aliments sur la table						
e. Mes proches, tels que ma famille immédiate, les autres membres de ma famille et/ou mes ami·e·s m’ont encouragé·e à suivre un régime à teneur réduite en sodium						

34. Dans quelle mesure êtes-vous d'accord ou en désaccord pour dire : « Les personnes qui comptent pour moi veulent que je limite ma consommation de sodium »? *(Sélectionnez une seule réponse)*

Tout à fait en désaccord	↔			Tout à fait d'accord	Je ne sais pas
1	2	3	4	5	

35. Plusieurs facteurs environnementaux peuvent influer sur la réduction de la quantité de sodium dans votre alimentation. Veuillez indiquer dans quelle mesure vous êtes d'accord ou en désaccord avec les affirmations suivantes, que vous essayiez ou non de limiter votre consommation de sodium. *(Sélectionnez une réponse dans chaque ligne.)*

	Tout à fait en désaccord	↔			Tout à fait d'accord	Ne s'applique pas
	1	2	3	4	5	
a. Lorsque je fais mon épicerie, je trouve que les produits à faible teneur en sodium ne sont pas disponibles ou que les choix sont peu variés						
b. Il est difficile de trouver des options à faible teneur en sodium sur les plateformes de vente et/ou de livraison de produits alimentaires en ligne						
c. Lorsque je mange dans des restaurants <u>à service rapide</u>, je trouve que les produits à faible teneur en sodium ne sont pas disponibles ou que les choix sont peu variés						
d. Lorsque je mange dans des restaurants <u>avec service à table</u> restaurants, je trouve que les produits à faible teneur en sodium ne sont pas disponibles ou que les choix sont peu variés						

36. Veuillez indiquer dans quelle mesure vous êtes d'accord ou en désaccord avec les affirmations suivantes concernant le contrôle que vous exercez sur votre consommation de sodium, que vous essayiez ou non de limiter votre consommation de sodium. *(Sélectionnez une réponse dans chaque ligne.)*

	Tout à fait en désaccord		↔		Tout à fait d'accord
	1	2	3	4	5
a. Je peux contrôler la quantité de sodium que je consomme					
b. C'est à moi de limiter ma consommation de sodium					
c. Si je le voulais, il me serait facile de limiter le sodium dans mon alimentation					
d. J'ai la volonté de réduire ma consommation de sodium					

37. Veuillez indiquer dans quelle mesure vous êtes d'accord ou en désaccord avec les affirmations suivantes, que vous essayiez ou non de limiter votre consommation de sodium. *(Sélectionnez une réponse dans chaque ligne.)*

	Tout à fait en désaccord ↔				Tout à fait d'accord	Ne s'applique pas
	1	2	3	4	5	
a. Je ne sais pas comment m'y prendre pour réduire la quantité de sodium que je consomme						
b. Il est difficile de comprendre les informations sur le sodium que l'on trouve sur les étiquettes des aliments						
c. J'ai confiance en ma capacité à préparer mes repas à la maison à partir de zéro						
d. J'ai confiance en ma capacité à identifier les sources de sodium dans mon alimentation						
e. Je suis sûr·e de pouvoir remplacer les aliments à teneur élevée en sodium par des aliments à faible teneur en sodium						

38. À quelle fréquence faites-vous <u>personnellement</u> l'épicerie dans votre foyer? *(Sélectionnez une seule réponse)*
a. Jamais
b. Rarement
c. Parfois
d. Très souvent
e. Toujours
f. Je préfère ne pas répondre

39. À quelle fréquence cuisinez-vous <u>personnellement</u> vos repas? *(Sélectionnez une seule réponse)*
a. Je cuisine tous mes repas
b. Je cuisine la majorité de mes repas
c. Je cuisine la moitié de mes repas
d. Je cuisine une partie de mes repas
e. Je ne cuisine aucun de mes repas
f. Je préfère ne pas répondre

40. Veuillez indiquer dans quelle mesure vous êtes d'accord ou en désaccord avec l'affirmation suivante: « Je choisis les produits alimentaires sur la base de mon expérience ou de mes connaissances et non en fonction des étiquettes alimentaires». *(Sélectionnez une seule réponse)*

Tout à fait en désaccord		↔		Tout à fait d'accord
1	2	3	4	5

41. La question suivante porte sur vos habitudes alimentaires au cours du dernier mois. *(Sélectionnez une seule réponse dans chaque ligne.)*

	Jamais	1 à 3 fois par mois	1 à 3 fois par semaine	4 à 6 fois par semaine	7 fois ou plus par semaine
Je mange de la nourriture provenant d'un restaurant. Cela comprend les restaurants avec salle à manger, les plats pour emporter ou en livraison et la restauration rapide.					

The** Abbreviated **Behavioural Assessment Instrument for dietary Sodium (BAIS-ab) - Factor model

This abridged version of the survey includes the key questions retained after psychometric analyses. These questions are organized into seven validated constructs representing major factors that influence sodium intake. The complete version of the survey is provided in Appendix A.

1. This is a warning label that Canadians will soon find on the front of some food packages. What does this label tell you about the food product? *(Check one)*

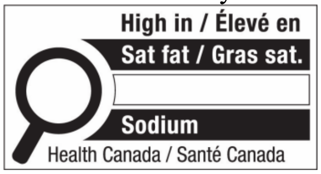

a. The food is *low* in sodium and saturated fat
b. The food is *high* in sodium and saturated fat
c. The food is *high* in saturated fat and *low* in sodium
d. The food *does not contain* sodium and saturated fat
e. I do not know

2. To the best of your knowledge, which of the following is the main source of sodium in the Canadian diet? *(Check one)*
a. Sodium added during cooking
b. Sodium added at the table
c. Sodium added to processed and prepared foods
d. Sodium that is naturally present in food
e. I do not know

3. Please tell us to what extent you agree or disagree with the following statements. *(Check one per line)*

	Strongly Disagree ↔ Strongly Agree				
	1	2	3	4	5
a. Kosher salt, sea salt and gourmet salts contain less sodium than regular table salt					
b. My sodium intake is low because I do not add salt to my food					
c. People with low or normal blood pressure do not need to be concerned with the amount of sodium in their diet					
d. Children do not need to limit their sodium intake					

4. To the best of your knowledge, how much sodium do you think Canadians eat? *(Check one)*
a. Far too much
b. Too much
c. The right amount
d. Too little
e. Far too little
f. I do not know

5. Below we have listed some of the common ways to lower the amount of sodium in your diet. In the past <u>month</u>, to what extent have you personally done any of the following, regardless of whether or not you are trying to limit your sodium? (*Check one per line*)

	Never do this ↔				Always do this	Not Applicable
	1	2	3	4	5	
a. Make your own soups, sauces, and salad dressings						
b. Eat more fresh fruits and vegetables						
c. Eat fewer packaged, ready-to-eat foods						
d. Limit or avoid eating food made in restaurants or cafeterias						
e. Read the Nutrition Facts table to determine if a product is high or low sodium						

6. When there are many products to choose from, we are interested to know how you would find the lowest sodium choice. To what extent do you/would you do the following in order to find the lowest sodium product? *(Check one per line)*

	Never do this ↔				Always do this
	1	2	3	4	5
a. Look at the total amount of sodium (mg) on the Nutrition Facts table					
b. Look at the % Daily Value (% DV) on the Nutrition Facts table					
c. Look for a symbol or a logo on the food package that suggests a product is a healthy choice					
d. Look for a message or claim on the food package that says a product is “low sodium”, “reduced sodium” or “sodium free”					
e. Look at the ingredients list for sodium sources					

7. Please tell us the extent to which you agree or disagree with the following statements. *(Check one per line)*

	Strongly Disagree ↔				Strongly Agree
	1	2	3	4	5
a. I think about sodium in my diet					
b. Reducing my sodium intake is important to me					
c. My health would improve if I lowered the amount of sodium I eat					

8. How concerned are you about the amount of sodium in your diet? *(Check one)*

a. Extremely concerned
b. Very concerned
c. Somewhat concerned
d. Not very concerned
e. Not at all concerned

9. Please tell us to what extent you agree or disagree with the following statements. *(Check one per line)*

	Strongly Disagree ↔				Strongly Agree
	1	2	3	4	5
a. I will likely be concerned about my sodium intake <u>later in my life, but not now</u>					
b. Even if eating too much sodium has negative health effects, I am willing to take the risk					

10. Please tell us the extent to which you agree or disagree with the following statements. *(Check one per line)*

	Strongly Disagree ↔				Strongly Agree	Not Applicable
	1	2	3	4	5	
a. Sodium makes food taste good						
b. I often have cravings for salty foods						
c. When I have feelings of stress or sadness, I comfort myself by eating foods that may be high in sodium						
d. I enjoy eating salty foods or snacks						

11. Please tell us the extent to which you agree or disagree with the following statement(s), regardless of whether or not you are trying to limit your sodium intake. *(Check one per line)*

	Strongly Disagree ↔				Strongly Agree	Not Applicable
	1	2	3	4	5	
a. I am willing to sacrifice taste in order to limit my sodium intake						

12. Please tell us the extent to which you agree or disagree with the following statements regarding your control over your sodium intake, regardless of whether or not you are trying to limit your sodium intake. *(Check one per line)*

	Strongly Disagree ↔				Strongly Agree
	1	2	3	4	5
a. I can control the amount of sodium I consume					
b. If I wanted to, it would be easy for me to limit the sodium in my diet					
c. I have willpower to reduce my sodium intake					

13. Please tell us the extent to which you agree or disagree with the following statements, regardless of whether or not you are trying to limit your sodium intake. *(Check one per line)*

	Strongly Disagree ↔				Strongly Agree	Not Applicable
	1	2	3	4	5	
a. I do not know how to reduce the amount of sodium I eat						
b. I am confident I can identify the sources of sodium in my diet						
c. I am confident I can replace high sodium foods with lower sodium foods						

Instrument Abrégé D'Évaluation Comportementale pour le Sodium alimentaire (BAIS-ab) – Modèle factoriel

Cette version abrégée du questionnaire comprend les questions clés retenues après les analyses psychométriques. Ces questions sont regroupées en sept construits validés représentant les principaux facteurs influençant l'apport en sodium. La version complète du questionnaire est fournie en Annexe A.

1. Ceci est une étiquette de mise en garde que les Canadien·ne·s trouveront bientôt sur le devant de certains emballages de produits alimentaires. Que vous apprend cette étiquette sur le produit alimentaire? *(Sélectionnez une seule réponse)*

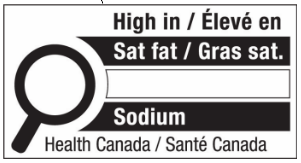

a. L'aliment a une *faible* teneur en sodium et en gras saturés
b. L'aliment a une teneur *élevée* en sodium et en gras saturés
c. L'aliment a une teneur *élevée* en gras saturés et faible en sodium
d. L'aliment *ne contient pas* de sodium ni de gras saturés
e. Je ne sais pas

2. À votre connaissance, laquelle des sources suivantes est la principale source de sodium dans l'alimentation des Canadiens? *(Sélectionnez une seule réponse)*
a. Sodium ajouté pendant la cuisson
b. Sodium ajouté à table
c. Sodium ajouté aux aliments transformés et préparés
d. Sodium naturellement présent dans les aliments
e. Je ne sais pas

3. Veuillez indiquer dans quelle mesure vous êtes d'accord ou en désaccord avec les affirmations suivantes. *(Sélectionnez une seule réponse chaque ligne)*

	Tout à fait en désaccord	↔			Tout à fait d'accord
	1	2	3	4	5
a. Le sel casher, le sel de mer et les sels de spécialité contiennent moins de sodium que le sel de table ordinaire					
b. Mon apport en sodium est faible parce que je n'ajoute pas de sel à mes aliments					
c. Les personnes dont la tension artérielle est basse ou normale n'ont pas à se préoccuper de la quantité de sodium dans leur alimentation					
d. Les enfants n'ont pas besoin de limiter leur consommation de sodium					

4. À votre avis, quelle quantité de sodium est consommée par les Canadien·ne·s? *(Sélectionnez une seule réponse)*
   a. Beaucoup trop
   b. Trop
   c. Juste la quantité qu'il faut
   d. Trop peu
   e. Beaucoup trop peu
   f. Je ne sais pas

5. Vous trouverez ci-dessous une liste des moyens les plus courants pour réduire la quantité de sodium dans votre alimentation. Au cours du dernier mois, dans quelle mesure avez-vous personnellement fait l'une des choses suivantes, que vous essayiez ou non de limiter votre consommation de sodium? (*Sélectionnez une seule réponse chaque ligne*)

	Je ne fais jamais ceci ↔ Je fais toujours ceci					Ne s'applique pas
	1	2	3	4	5	
a. Préparer vous-même vos soupes, sauces et vinaigrettes						
b. Manger plus de fruits et de légumes frais						
c. Manger moins d'aliments emballés et prêts à consommer						
d. Limiter ou éviter de manger des aliments préparés dans les restaurants ou cafétérias						
e. Lire le tableau de la valeur nutritive pour déterminer si un produit a une teneur en sodium faible ou élevée						

6. Lorsque vous avez le choix entre de nombreux produits, nous aimerions savoir comment vous vous y prenez pour trouver le choix qui contient le moins de sodium. Dans quelle mesure faites-vous ou feriez-vous ce qui suit afin de trouver le produit qui a la plus faible teneur en sodium? *(Sélectionnez une seule réponse chaque ligne)*

	Je ne fais jamais ceci ↔ Je fais toujours ceci				
	1	2	3	4	5
a. Vérifier la teneur totale en sodium (mg) dans le tableau de la valeur nutritive					
b. Vérifier le % de valeur quotidienne (% VQ) dans le tableau de la valeur nutritive					
c. Regarder sur l'emballage du produit alimentaire s'il y a un symbole ou un logo qui suggère que le produit est un choix sain					
d. Regarder sur l'emballage du produit alimentaire s'iI y a un message ou une allégation qui indique que le produit est « faible en sodium », « à teneur réduite en sodium » ou « sans sodium »					
e. Vérifier les sources de sodium dans la liste des ingrédients					

7. Veuillez indiquer dans quelle mesure vous êtes d'accord ou en désaccord avec les affirmations suivantes. *(Sélectionnez une réponse dans chaque ligne.)*

	Tout à fait en désaccord ↔				Tout à fait d'accord
	1	2	3	4	5
a. Je tiens compte du sodium dans mon alimentation					
b. Il est important pour moi de réduire ma consommation de sodium					
c. Ma santé s'améliorerait si je réduisais la quantité de sodium que je consomme					

8. Dans quelle mesure êtes-vous préoccupé·e par la quantité de sodium dans votre alimentation? *(Sélectionnez une seule réponse)*
   a. Extrêmement préoccupé·e
   b. Très préoccupé·e
   c. Assez préoccupé·e
   d. Peu préoccupé·e
   e. Pas du tout préoccupé·e

9. Veuillez indiquer dans quelle mesure vous êtes d'accord ou en désaccord avec les affirmations suivantes. *(Sélectionnez une seule réponse chaque ligne)*

	Tout à fait en désaccord ↔				Tout à fait d'accord
	1	2	3	4	5
a. Je m'inquièterais probablement de ma consommation de sodium <u>plus tard dans ma vie, mais pas maintenant</u>					
b. Même si le fait de consommer trop de sodium a des effets négatifs sur ma santé, je suis prêt·e à en prendre le risque					

10. Veuillez indiquer dans quelle mesure vous êtes d'accord ou en désaccord avec les affirmations suivantes. *(Sélectionnez une réponse dans chaque ligne)*

	Tout à fait en désaccord ↔				Tout à fait d'accord	Ne s'applique pas
	1	2	3	4	5	
a. Le sodium donne du goût aux aliments						
b. J'ai souvent des envies d'aliments salés						
c. Lorsque je ressens du stress ou de la tristesse, je me réconforte en mangeant des aliments qui peuvent être riches en sodium						
d. J'aime manger des collations ou des aliments salés						

11. Veuillez indiquer dans quelle mesure vous êtes d’accord ou en désaccord avec les affirmations suivantes, que vous essayiez ou non de limiter votre consommation de sodium. *(Sélectionnez une réponse dans chaque ligne.)*

	Tout à fait en désaccord ↔ Tout à fait d'accord					Ne s'applique pas
	1	2	3	4		
a. Je suis prêt·e à sacrifier le goût pour limiter ma consommation de sodium						

12. Veuillez indiquer dans quelle mesure vous êtes d’accord ou en désaccord avec les affirmations suivantes concernant le contrôle que vous exercez sur votre consommation de sodium, que vous essayiez ou non de limiter votre consommation de sodium. *(Sélectionnez une réponse dans chaque ligne.)*

	Tout à fait en désaccord ↔ Tout à fait d'accord				
	1	2	3	4	5
a. Je peux contrôler la quantité de sodium que je consomme					
b. Si je le voulais, il me serait facile de limiter le sodium dans mon alimentation					
c. J'ai la volonté de réduire ma consommation de sodium					

13. Veuillez indiquer dans quelle mesure vous êtes d’accord ou en désaccord avec les affirmations suivantes, que vous essayiez ou non de limiter votre consommation de sodium. *(Sélectionnez une réponse dans chaque ligne.)*

	Tout à fait en désaccord ↔ Tout à fait d'accord					Ne s'applique pas
	1	2	3	4	5	
a. Je ne sais pas comment m’y prendre pour réduire la quantité de sodium que je consomme						
b. J’ai confiance en ma capacité à identifier les sources de sodium dans mon alimentation						
c. Je suis sûr·e de pouvoir remplacer les aliments à teneur élevée en sodium par des aliments à faible teneur en sodium						
